# Paracrine Effects of Recombinant Human Adiponectin Promote Bone Regeneration

**DOI:** 10.3389/fcell.2021.762335

**Published:** 2021-11-01

**Authors:** Yanping Gong, Yang Wang, Yiqing Zhang, Liangchen Wang, Lijuan Wan, Yuan Zu, Chunlin Li, Xin Wang, Zhong-Kai Cui

**Affiliations:** ^1^Department of Endocrinology, The Second Medical Center, National Clinical Research Center for Geriatric Disease, The Chinese People’s Liberation Army General Hospital, Beijing, China; ^2^Department of Cell Biology, School of Basic Medical Sciences, Southern Medical University, Guangzhou, China; ^3^Guangdong Provincial Key Laboratory of Bone and Joint Degeneration Diseases, The Third Affiliated Hospital, Southern Medical University, Guangzhou, China; ^4^Institute of Orthopedics, The First Medical Center, The People’s Liberation Army General Hospital, Beijing, China; ^5^Bioland Laboratory, Guangzhou, China

**Keywords:** adiponectin, bone formation, paracrine effect, AdipoR1, medulla injection

## Abstract

Bone regeneration is a delicate physiological process. Non-union and delayed fracture healing remains a great challenge in clinical practice nowadays. Bone and fat hold a close relationship to remain balanced through hormones and cytokines. Adiponectin is a well-known protein to maintain the hemostasis, which may be an interesting target for fracture healing. Herein, we provided a facile and efficient method to obtain high-purity and high-yield recombinant human adiponectin (ADPN). The biocompatibility and the pharmaceutical behaviors were evaluated in Sprague–Dawley rats. The paracrine effects of adiponectin on bone fracture healing were investigated with a rat tibia fracture model *via* intrabone injection. Significantly accelerated bone healing was observed in the medulla injection group, indicating the paracrine effects of adiponectin could be potentially utilized for clinical treatments. The underlying mechanism was primarily assessed, and the expression of osteogenic markers, including bone morphogenic protein 2, alkaline phosphatase, and osteocalcin, along with adiponectin receptor 1 (AdipoR1), was markedly increased at the fracture site. The increased bone healing of ADPN treatment may result from both enhanced osteogenic proliferation as well as differentiation. Cell experiments confirmed that the expression of osteogenesis markers increased significantly in ADPN treatment groups, while it decreased when the expression of AdipoR1 was knocked down by siRNA. Our study provided a feasible and efficacious way for bone fracture treatment with local administration of ADPN, which could be rapidly translated into the clinics.

## Introduction

Bone regeneration is a delicate and complex physiological process (Clarke, 2008). Bone defects resulted from traumatic injury, tumor resection, and degenerative diseases are challenging problems in clinics (Burge et al., 2007). The gold standard in clinical practice is an autologous bone graft (Einhorn and Gerstenfeld, 2015). Unfortunately, the limited sources, the injury of the donor site and perioperative complications significantly restrict the employment of this approach (Baqain et al., 2009). Bone morphogenic protein 2 (BMP-2) is considered the most efficacious cytokine for bone repair and has been extensively studied for the treatment of various bone fractures and bone defects (Glassman et al., 2008; Long, 2011). However, supraphysiological dosage is necessary in clinical practice, causing undesirable side effects, including hollow bone formation, life-threatening tissue edema, and cancer (Cahill et al., 2009; Barbour et al., 2011; Skovrlj et al., 2015). In addition, the high cost for BMP-2 becomes a heavy financial burden for the health system. Therefore, developing alternative strategies are imperative for bone regeneration.

The close relationship between bone and fat formation was well acknowledged in the literature (Gimble et al., 2006). The bone marrow mesenchymal stem cells may take different pathways during their lifetime, to differentiate and transdifferentiate in response to changes in the microenvironment to bone or fat (Chen et al., 2016). The inverse relationship between bone and fat implied that agents inducing adipogenesis inhibited osteoblast differentiation and *vice versa* (Naot et al., 2017). These results coincided with classic pathological and epidemiological phenomena of increased marrow adiposity with aging and bone loss.

Human adiponectin is a 30-kDa adipose-derived secreted protein containing 244 amino acid residues, with an N-terminal signal sequence, a hypervariable region, a collagenous domain, and a globular domain (Scherer et al., 1995). Since it was first discovered in 1995, efforts have been devoted to unraveling the biological activities of adiponectin. Metabolic regulation and maintenance of whole-body energy homeostasis are recognized as the main physiological role of adiponectin (Wang and Scherer, 2016; China et al., 2018). Anti-inflammatory and antiapoptotic effects were demonstrated as major physiological activities of adiponectin as well (Ohashi et al., 2010). Adiponectin binds to two seven-transmembrane domain receptors, AdipoR1 and AdipoR2. Interestingly, unlike the well-known G-protein-coupled receptors, the N-terminus is located inside the cell, whereas the C-terminus faces outward for both AdipoR1 and AdipoR2. AdipoR1 was found abundant in skeletal muscle and the liver *via* ubiquitous expression, while AdipoR2 was isolated mostly from the liver (Kang et al., 2009). T-cadherin, highly expressed in endothelial and smooth muscle cells, was identified as a third adiponectin receptor (Matsuda et al., 2015).

In light of the inverse relationship between serum adiponectin levels and fat mass, the inverse relationship between bone marrow fat and bone mass inspired researchers to focus on the effects of adiponectin on bone regeneration. *In vitro*, adiponectin was reported to increase the mRNA expression of alkaline phosphatase (ALP) in preosteoblasts and promote the mineralization of the bone matrix (Williams et al., 2009; Naot et al., 2017). Furthermore, in a mouse model, the elevated adiponectin in the bloodstream significantly increased the volume of cancellous bone (China et al., 2017). Interestingly, an inverse correlation was demonstrated in clinical studies between serum adiponectin concentrations and bone mineral density (BMD) (Napoli et al., 2010). AdipoR1 and AdipoR2 were found to be expressed in primary human osteoblasts and in bone marrow macrophages, which could be the possible reasons for adiponectin playing a significant role in bone regeneration (Berner et al., 2004; Wu et al., 2019). Contradictory results in the literature were demonstrated as well; therefore, more evidence is needed to further clarify the physiological role of adiponectin in bone biology.

Although the endocrine effects of the secreted protein adiponectin from adipose tissue into the circulation account for the energy homeostasis, its local paracrine effects may play a pivotal role in bone regeneration (Martinez-Huenchullan et al., 2020). Here, we recombined human globular adiponectin (ADPN) and further characterized the pharmacokinetic behaviors and toxicity through medulla injection. A rat tibia fracture model was exploited to evaluate the capability of ADPN for bone repair. In addition, we attempted to unravel the underlying mechanism of adiponectin promoting bone regeneration.

## Materials and Methods

### Materials

Glutamine synthetase (GS), methionine sulfoximine (MSX), Chinese hamster ovary K1 (CHO-K1) cells, Dulbecco’s modified Eagle’s medium (DMEM), fetal bovine serum (FBS), penicillin/streptomycin (P/S), insulin, and pentobarbital sodium were supplied by Sigma-Aldrich (St. Louis, MO, United States). Biotin-conjugated monoclonal antibodies for ELISA were purchased, including osteoprotegerin (OPG) ab255723, ADPN ab108784 (Abcam, Cambridge, United Kingdom). Antibodies for Western blot were GAPDH ab8245, AdipoR1 ab70362, BMP-2 ab14933 (Abcam, Cambridge, United Kingdom), and ALP sc-271431 and Osteocalcin (OCN) sc-376726 (Santa Cruz, CA, United States). Antibodies used for immunofluorescent staining were BMP-2 ab6285 (Abcam, Cambridge, United Kingdom); ALP sc-271431, OCN sc-390877 (Santa Cruz, CA, United States); and AdipoR1 BM4566 (Boster Bio, CA, United States).

### Methods

#### Evaluation of Pharmacokinetics and Toxicity

All animal experiments were performed in accordance with the guidelines of the Ethics Committee of the Chinese People’s Liberation Army General Hospital, Beijing, China. Sprague–Dawley male rats (*n* = 15, 8 weeks old) were randomly divided into three groups, which were treated with 0, 1, and 2 mg/kg of recombinant ADPN in PBS *via* medulla injection (G1, G2, and G3, respectively). Blood samples were collected in 1% heparin tubes *via* fundus vein plexus at predetermined time points. The supernatant was obtained by centrifugation at 3,000 rpm for 10 min, and ADPN concentration in serum was assessed with the ELISA kit (m1061301-3, Mlbio, Shanghai, China), following the protocol of the manufacturer. An automated enzymatic procedure (Cobas E601, Roche, Basel, Switzerland) was employed for blood biochemistry evaluation. Sysmex XE22100 automatic blood analyzer was used for blood routine examination. Organs including hearts, livers, spleens, lungs, kidneys, brains, and pancreases were harvested at the end time point. After weighing, all the organs were fixed in 4% paraformaldehyde, embedded in paraffin, and sectioned at the thickness of 5 μm. H&E staining (G1120, Solarbio, Beijing, China) was carried out for all the sections to evaluate the toxicity.

#### Tibia Fracture Model

Sprague–Dawley male rats (8 weeks old) were anesthetized with 3 ml/kg of 3% pentobarbital sodium *via* intraperitoneal injection. A scalpel blade (#15) was used to open the knee joint. A 20-gaged syringe was inserted into the medulla of the tibia for drug injection. A Kirshner needle (1 mm) was inserted into the distal tibia at a penetration depth of about 22 ± 2 mm. The excess proximal needle was cut off with a bone cutter. Three-point forceps were fixed to the left leg. The forceps were closed until a crack was heard, and the resistance of the forceps suddenly dropped. After surgery, all animals were allowed to recover on a warm sheet and then transferred to the vivarium for postoperative care. In preparation for the operative treatment, all animals received analgesia with subcutaneous injections of buprenorphine at a concentration of 0.1 mg/kg for 3 days. To prevent potential infection, all animals received 80,000 U of penicillin *via* intramuscular injection for 3 days.

#### Microcomputerized Tomography Scanning

Animals were imaged, at weeks 2, 4, and 6, using a high-resolution microcomputerized tomography (μCT) (Quantum GX μCT System, PerkinElmer, Waltham, MA, United States) with 90 kV, 80 μA, and 4.5-μm resolution. Visualization and reconstruction of the data were obtained using the Quantum GX μCT Workstation imaging software (PerkinElmer, Waltham, MA, United States). The volume of interest was defined manually as follows: The cortex area was defined as the region enclosed by the callus and cortical boundaries in tomograms. The trabecular area was an irregular and anatomic region of interest drawn manually, a few voxels away from endocortical surface to medullary space. The cortical pad area (CT. Ar) and cortical pad thickness (CT. Th), BMD, bone volume density (BV/TV, %) and mean thickness of the trabecular (Tb. Th), trabecular number (Tb. N, mm^–1^), trabecular separation (Tb. Sp), structure model index (SMI), and bone density of connection (Conn. D, mm^–1^) were derived using the Analyze software (AnalyzeDirect, KS, United States).

#### Mechanical Evaluation

Six weeks post surgery, rats were euthanized, and tibias were harvested and undergone three-point flexural mechanical testing on the biomechanics machine (MTS 858, MTS, United States). An axial force of 5 N was preloaded to the bone, and constant linear propulsion (5 mm min^–1^) was applied to a lever arm attached to one of the pivoted axes to provide a uniform movement.

#### Histological Evaluation

The harvested tibias were fixed in 4% paraformaldehyde for 48 h, followed by decalcification in 10% EDTA solution under gentle shaking for 4 weeks. The EDTA solution was changed every 2 days. Decalcified samples were embedded in paraffin and cut into 5-μm-thick sections. The tissue sections were deparaffinized and stained with H&E.

Masson trichrome staining (G1340, Solarbio, Beijing, China) was also performed to detect new bone formation. The blue color, indicative of new or mature bone, was observed using an Olympus U-RFL-T microscope. Additional sections underwent immunohistochemical analysis. The deparaffinized sections were processed with citric acid for antigen retrieval and thereafter incubated with the primary antibody BMP-2 (1:400 dilution), AdipoR1 (1:400 dilution), ALP (1:200 dilution), and OCN (1:200 dilution) and were detected by the HRP/DAB kit (Beyotime, Beijing, China). The sections were further counterstained with Mayer’s hematoxylin (Beyotime, Beijing, China).

#### Protein Quantification

The expression of ALP, BMP-2, OCN, and AdipoR1 were examined with Western blot, and the normalized values of the blots were quantified with imageJ. OPG in the serum was quantified with ELISA.

Callus tissue (approximately 5 mm) around each bone fracture position was collected, weighed, and transferred into 1.5-ml Eppendorf tubes. RIPA buffer (6 μl/mg) and 0.174 mg/ml of PMSF (benzyl sulfonyl fluoride, pyrolysis liquid with PMSF, 100:1 v/v) were added into each tube. Proteins were extracted, and Bio-Rad Dc protein assay (Bio-Rad, Hercules, CA) was carried out to determine the protein concentration for further Western blot experiments.

#### Knockdown of AdipoR1 by siRNA

BMSCs (P3) were inoculated in 12-well plates at a cell density of 1 × 10^4^ cells/well. AdipoR1 siRNA (0.8 μg) was diluted with 50 μl of DMEM, and 2 μl of Lipofectamine 2000 (SolarBio China, Beijing, YZ-11668) was diluted with 50 μl of DMEM, and incubated for 5 min at room temperature. The transfection reagent and AdipoR1 siRNA diluent were mixed, and the complex was added to the well plate and incubated for 24 h. The transfection was performed on the first and fourth day, respectively.

#### Immunofluorescent Images of Bone Marrow Mesenchymal Stem Cells

BMSCs (P3) were inoculated in 12-well plates at a cell density of 1 × 10^4^ cells/well. ADPN (10 μg/ml) and the control group without drugs were added accordingly. The medium was replaced every other day for 7 days. The wells were rinsed with PBS three times. Four percent neutral paraformaldehyde was added. Fifteen minutes later, 0.1% Triton X-100 was applied to lysate the cells for 15 min. Five percent goat serum was used for blocking. Drops of primary antibodies were added (the same antibodies as in animal experiments) with dilution concentrations of 1:100 and incubated overnight in a wet box in a refrigerator at 4°C. Secondary antibody (antibody dilution: 1:200, ZSGB-Bio, China Beijing, Alexa Fluor^®^ 488, ZF-0512, Alexa Fluor^®^ 594 ZF-0513) was added, and the nuclei were stained with DAPI.

#### Statistical Analysis

Data were presented as mean ± standard deviation. Multiple comparisons were assessed using the one-way or two-way analysis of variance (ANOVA). The analysis of variances followed by the *Tukey’s hoc* test was employed in this work, and *p* < 0.05 was considered statistically significant.

## Results

### Amplification and Identification of Human Globular Adiponectin

Natural human adiponectin fragment (∼748 bp) was successfully amplified from cDNA sequence after optimizing codons. The size of adiponectin-Fc-GS fragment was around ∼1,434 bp. Two-way sequencing results confirmed that the sequence of inserted gene was identical to human globular adiponectin gene. After screening and purification, the recombinant human ADPN protein migrated at around 60 kDa by SDS-PAGE electrophoresis. ELISA was carried out to quantify the collected protein, and the yield was 20 μg/ml. The obtained ADPN was stored in glass vials after lyophilization. Details are described in Supplementary Figure 1.

### Pharmacokinetics of Adiponectin Based on Medulla Injection

**FIGURE 1:**
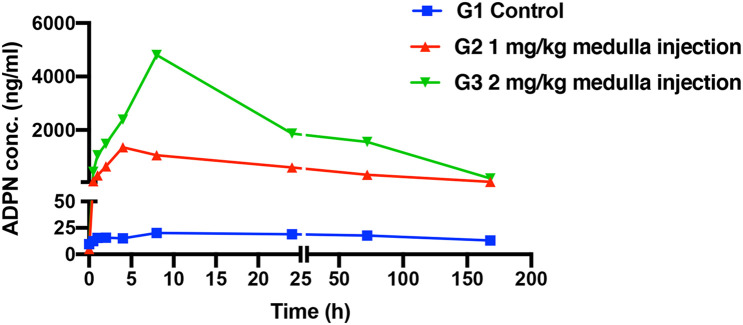
The serum concentrations of adiponectin (ADPN) based on medulla injections, *n* = 5.

The pharmacokinetic profiles of ADPN after medulla injection were plotted (Figure 1), and the calculated pharmacokinetic parameters are listed in Table 1. For G3 and G2, T_*max*_, the time to reach the maximum concentration (C_*max*_), was 8 and 4.8 h, and C_*max*_ was 4.9 ± 0.8 and 1.7 ± 0.3 μg/ml, respectively. The area under the curve (AUC) showed that ADPN exposure was significantly higher in G3 than that in G2 (*p* < 0.01). No significant difference was observed with the half-life of ADPN in the plasma between the two groups. The pharmacokinetic results indicated that most of ADPN could remain in the bone marrow *via* medulla injection.

**TABLE 1:** Pharmacokinetic parameters for adiponectin (ADPN) in SD rats.

**Parameters**	**Unit**	**Medulla injection**	
		**1 mg/kg**	**2 mg/kg**
Ke	h^–1^	0.02 ± 0.01	0.02 ± 0.01
T_1__/__2_	H	50.98 ± 22.51	54.24 ± 24.79
T_max_	H	4.80 ± 1.79*	8.00 ± 0.00
C_max_	μg⋅L^–1^	1,693.64 ± 302.30*	4,869.57 ± 825.30
AUC_0__–__*t*_	h⋅μg⋅L^–1^	3,158.48 ± 1,812.61**	79,483.18 ± 63,165.40
AUC_0__–__8_	h⋅μg⋅L^–1^	48,520.51 ± 89,123.65**	88,637.29 ± 68,594.26
Vd	L⋅kg^–1^	1,103.49 ± 596.23	532.71 ± 161.80
MRT	h	45.72 ± 5.56*	50.39 ± 5.20

*p < 0.05, **p < 0.01, G2 vs. G3.

### Toxicity of Adiponectin Based on Medulla Injection

**FIGURE 2:**
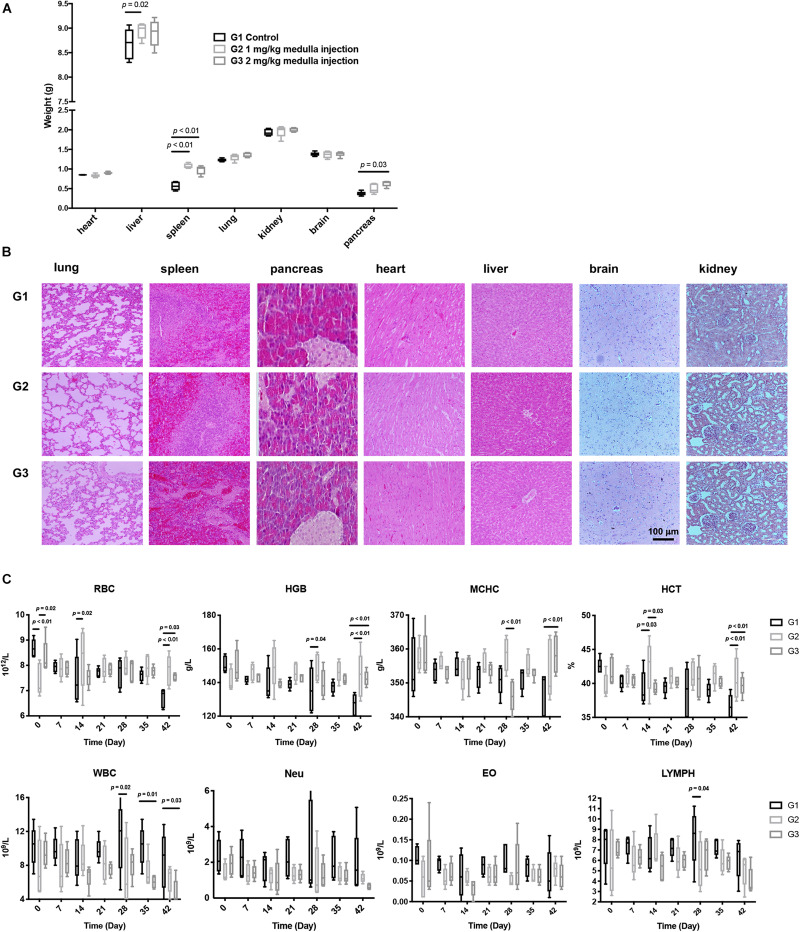
Toxicological experiments of ADPN based on local medulla injection. **(A)** The weight of various organs. **(B)** H&E staining images of various organs, scale bar = 100 μm. **(C)** The changes of red blood cell (RBC), hemoglobin (HGB), mean corpuscular hemoglobin concentration (MCHC), hematocrit (HCT), eosinophilic granulocyte, lymphocyte, and leukocyte levels after ADPN administration among the G1, G2, and G3 groups, *n* = 5.

All rats showed distinct difficulty in motion with their left legs right after surgery. Such symptom was alleviated a few hours later, yet four rats in each group still exhibited mild confined activity. All rats could move freely 24 h post surgery, with no abnormalities in the hair, canthus secretion, anus, genital, feces, behaviors, eating, drinking, etc., Organs, including hearts, livers, spleens, lungs, kidneys, pancreases, and brains, were harvested and weighed. Compared with controls, rats receiving ADPN presented significantly heavier spleens and pancreases (Figure 2A). H&E staining for all harvested organs was carried out to further examine the histomorphological variations (Figure 2B). No obvious changes were observed in all the organs among the three groups, indicating no direct toxicity of ADPN.

Hematology and serum biochemistry tests were carried out to further evaluate the biocompatibility of ADPN *in vivo*. RBC (red blood cell), HGB (hemoglobin), MCHC (mean corpuscular hemoglobin concentration), and HCT (hematocrit) in the ADPN treatment groups (G2 and G3) were significantly increased at day 42 compared with that in the control group postinjection. In contrast, eosinophilic granulocytes, lymphocytes, and leukocytes showed no remarkable alteration (Figure 2C). Hepatorenal function, blood glucose, triglyceride, high-density lipoprotein, and uric acid levels demonstrated no obvious changes for the investigated period (Supplementary Figure 2).

Rat bone marrow mesenchymal stem cells (BMSCs) were isolated and treated with different concentrations of ADPN (0, 1, 5, 10, 30 μg/ml) *in vitro*. CCK-8 assay was employed to plot the cell viability profiles with time progression. Enhanced proliferation was observed among all the treatment groups up to 48 h. Non-toxicity was present in all the groups for 72 h (Supplementary Figure 3).

### Osteogenic Ability of Adiponectin in Rat Tibia Fracture Model

**FIGURE 3:**
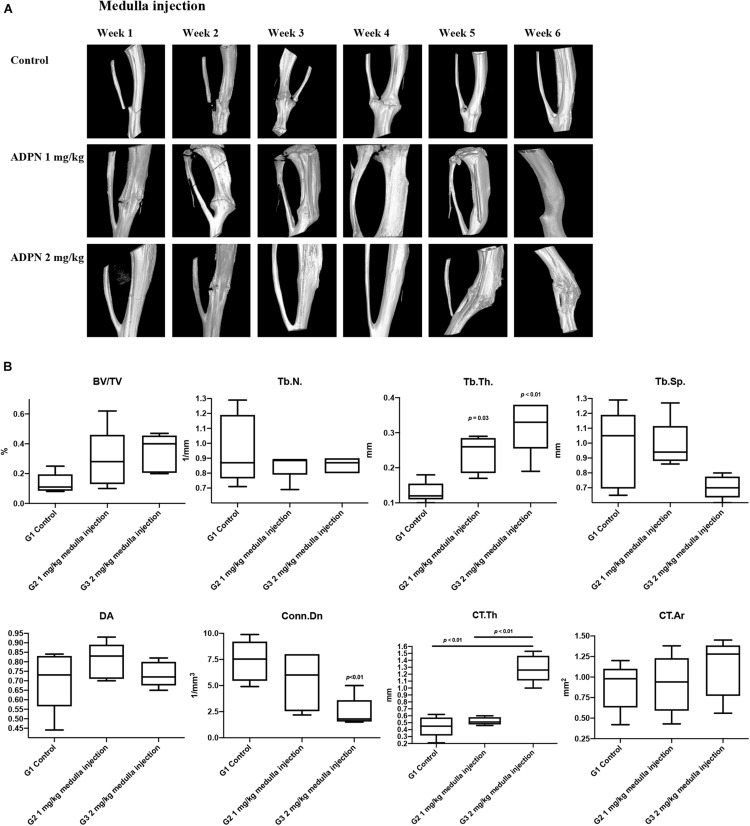
Local medulla injection of ADPN accelerated fracture healing and significantly influenced bone microarchitecture. **(A)** The three-dimensional μCT reconstruction of rat tibia from week 1 to 6. **(B)** Bone morphometric indices extracted from microcomputerized tomography (μCT) at week 6, *n* = 6.

*In vivo* high-resolution μCT was employed to evaluate the status of bone healing. ADPN treatment significantly improved callus formation after fracture. The three-dimensional μCT reconstruction analyses were carried out 6 weeks after surgery. The images delineated the recovery progress of tibial continuity gradually with time. With no treatment in the control group, the tibia healed 6 weeks postsurgery. The administration of ADPN *via* medulla injection remarkably shortened the recovery time. Especially, the higher dosage of ADPN (2 mg/kg) in G3 accelerated the healing within 3 weeks. For G2, 1 mg/kg of ADPN was administrated, and comparable healing was observed at week 4. Interestingly, as observation continued in G3 for 6 weeks, heterotrophic hyperplasia was observed in the μCT scans of some rats, even to the extent of the non-fractured fibula (Figure 3A).

Trabecular bone morphometric indices, including the bone volume fraction (BV/TV, %), trabecular number (Tb. N., mm^–1^), trabecular thickness (Tb. Th., μm^–1^), trabecular separation (Tb. Sp., μm^–1^), degree of anisotropy (DA), and connectivity density (Conn. Dn., mm^–3^) were extracted from the μCT images to evaluate the trabecular bone microarchitecture. The Tb. Th rose to 0.24 and 0.32 mm^–1^ for G2 and G3, respectively, considerably higher compared with that of the control group (0.13 mm^–1^). However, significant decrease in Conn. Dn. was observed for G3 to 2.42 from 7.47 mm^–3^ in the control group. Comparable values were derived for other extracted indices (Figure 3B).

Cortical bone morphometric indices, including cortical bone area (Ct. Ar., mm^2^) and average cortical thickness (Ct. Th., mm), were derived to assess the cortical bone microarchitecture. The Ct. Th. was markedly increased to 1.28 mm for G3, while comparable values of 0.45 and 0.52 were obtained for G1 and G2. Hyperplasia was obvious in G3, even extended to the fibula, which may contribute to the elevated Ct. Th. value.

**FIGURE 4:**
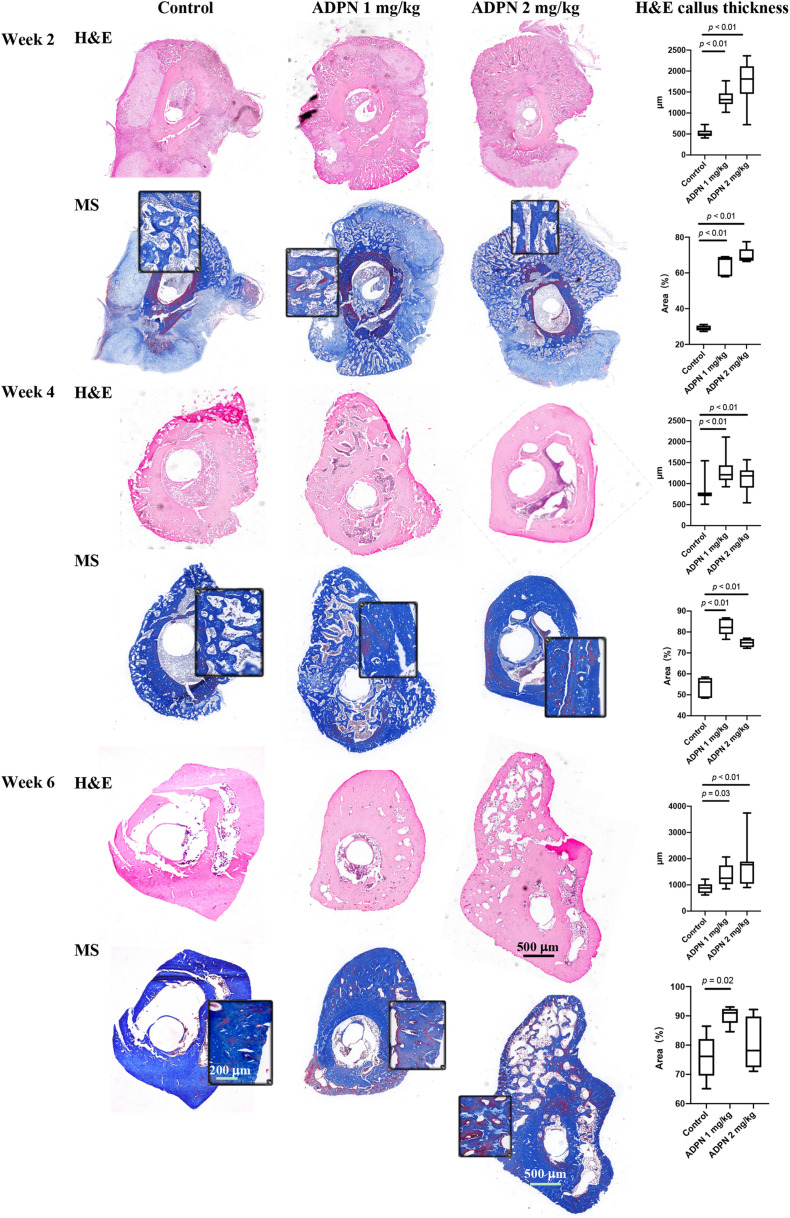
Histology evaluation of bone regeneration. The hematoxylin and eosin (H&E) and Masson trichrome staining (MS) images of rat tibia transverse sections at weeks 2, 4, and 6 (*n* = 5), scale bar = 500 μm and scale bar = 200 μm in the magnified images. The ADPN treatment groups showed thicker cortical bone and bone calluses. The callus thickness and the blue-stained area, indicative of new bone formation, were quantified and plotted.

The quality of new bone was further evaluated by histological evaluation with hematoxylin and eosin (H&E), and Masson trichrome staining (MS) (Figure 4). Immature and non-calcified calluses occupied an obvious larger area in the ADPN-treated groups, compared with the control group with time progression. The thickness of internal and external callus, and the quantitative values were plotted. Twelve directions evenly distributed from the center of the ring to the edge on each section were selected to measure the thickness. At week 6, the space between calluses increased in the control group, and abnormal hyperplasia was found in the 2 mg/kg ADPN group. Bone cortex became thicker, and relatively large gaps existed between multilayer immature bone calluses.

The blue-stained area in Masson’s trichrome staining was measured to evaluate the changes in bone regeneration. At week 2, hematoma and granulation tissue were mainly found in the treatment group, with little bone formation. The quality of bone tissue in the ADPN treatment group was better than that in the control group; however, no significant difference was observed between groups. At week 4, the hematoma was almost absorbed, and the callus became smaller with the regeneration of bone tissue. The quality of bone tissue in the treatment group was significantly better than that in the control group. At week 6, abnormal bone formation was found in the ADPN 2 mg/kg group. The MS section showed less blue staining, probably due to the formation of heterotopic hyperplasia. The quality of the new generated bone with ADPN 1 mg/kg treatment on week 6 was satisfactory with the blue stain area > 90%.

**FIGURE 5:**
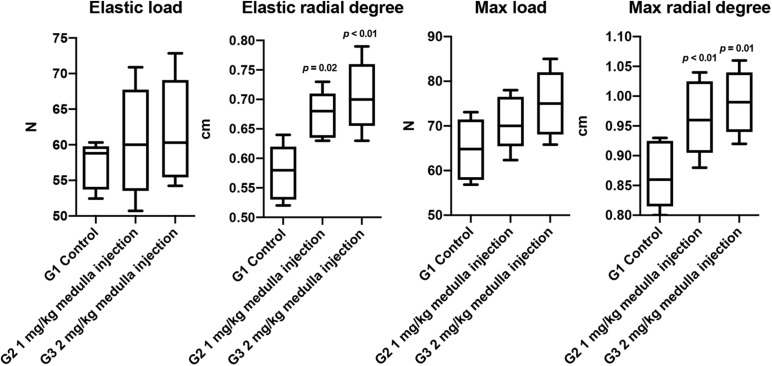
The biomechanical properties of rat tibia at week 6, *n* = 6.

The biomechanical properties of the regenerated bone, including elastic load, elastic radial degree, max load, and max radial degree, were evaluated (Figure 5). Significant improvements were observed in structural biomechanics of the healed tibia in the ADPN-treated groups, as well as in the elastic radial degree, maximal radial degree, and maximum load, especially in the ADPN 2 mg/kg group.

### Potential Mechanism of Adiponectin Promoting Fracture Healing

**FIGURE 6:**
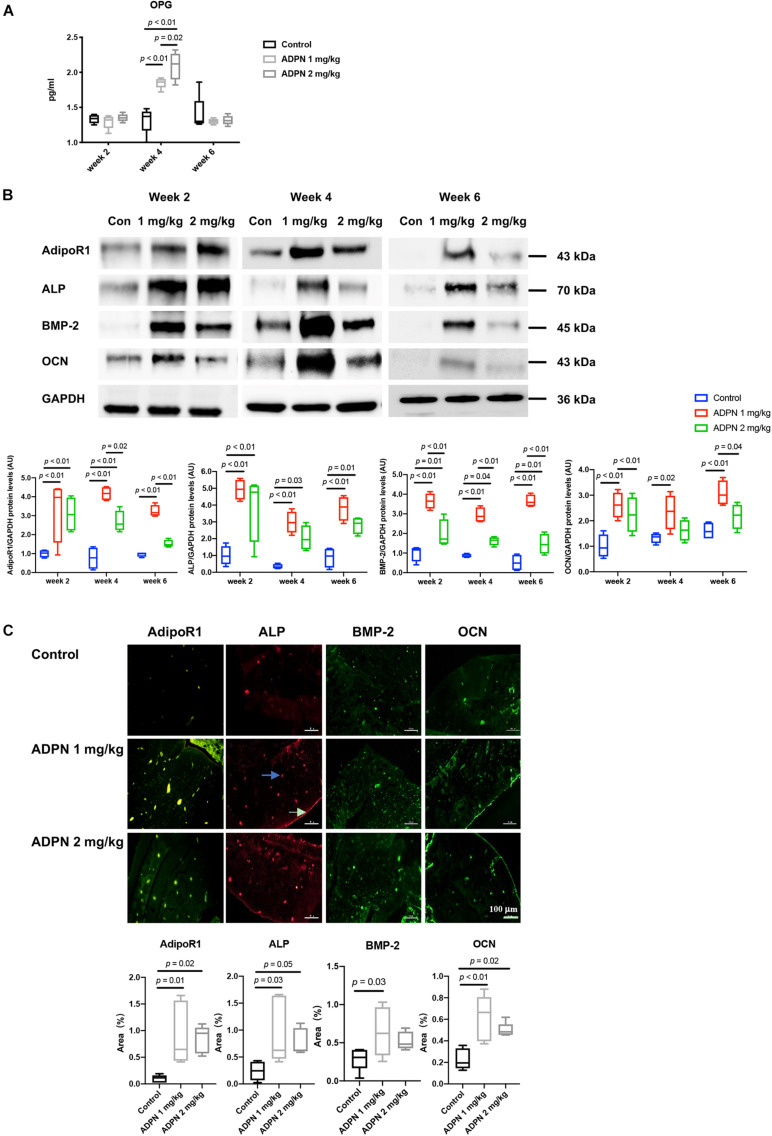
Mechanisms for local medulla injection of ADPN promoting bone healing. **(A)** Serum Osteoprotegerin (OPG) levels at weeks 2, 4, and 6. **(B)** WB analysis of alkaline phosphatase (ALP), bone morphogenic protein 2 (BMP-2), osteocalcin (OCN), and adiponectin receptor 1 (AdipoR1) expressions with corresponding quantification, **(C)** Immunofluorescent staining of ALP, OCN, BMP-2, and AdipoR1 in G2 (ADPN 1 mg/kg) and G3 (ADPN 2 mg/kg), and the corresponding quantification, *n* = 5. The white arrow indicates the periosteum, and the blue arrow indicates the lacuna, scale bar = 100 μm.

Osteoprotegerin (OPG) in the serum was considerably elevated after administration of ADPN. OPG is a well-known decoy receptor for RANKL, and OPG can inhibit RANK–RANKL interactions, resulting in suppressing osteoclastogenesis and bone resorption. The expression of OPG peaked at week 4 in both treatment groups. OPG level was significantly higher in the ADPN-treated groups than that in the control group (Figure 6A).

Western blot analysis showed that the maximum level of the early osteogenic marker ALP appeared at week 2 post surgery in both treatment groups, and its expression decreased with time progression. The BMP-2 and late osteogenic marker OCN were peaked at week 4 for the ADPN 1 mg/kg group. The ADPN 1 mg/kg treatment group exhibited significantly higher expressions of all three osteogenic markers, ALP, BMP-2, and OCN, than that of the ADPN 2 mg/kg at the three investigated time points (Figure 6B). The ADPN receptor AdipoR1 was also quantified with high expression in both the treatment groups at week 2 and markedly lower values at weeks 4 and 6 for the G3 than that of G2. Immunofluorescent staining images demonstrated that AdipoR1, ALP, BMP-2, and OCN were highly expressed during fracture healing following ADPN treatment (Figure 6C).

**FIGURE 7:**
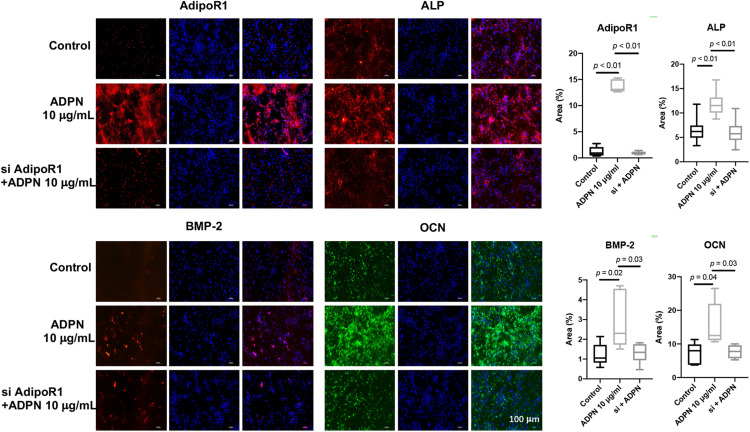
The immunofluorescent staining of ALP, OCN, BMP-2, and AdipoR1 on BMSCs with ADPN 10 μg/ml and AdipoR1 siRNA + ADPN 10 μg/ml, and the corresponding quantification, scale bar = 100 μm.

Rat BMSCs were treated with 10 μg/ml of ADPN and the AdipoR1 siRNA + 10 μg/ml of ADPN to evaluate the effects on osteogenesis. The addition of ADPN significantly elevated the expression of AdipoR1 as shown with the intensified red color in the image (Figure 7). After transfecting the AdipoR1 siRNA, the expression of AdipoR1 was remarkably knocked down. The osteogenic markers ALP, BMP-2, and OCN were significantly increased with the treatment of ADPN, while when knocking down of AdipoR1 with siRNA, the expression of those osteogenic markers declined concomitantly.

## Discussion

Bone regeneration and bone fracture healing remain great challenges in daily clinical practice. Alternative therapies are being developed, including the FDA-approved BMP-2 and BMP-7 treatments (Jo et al., 2015), to overcome the adverse effects of autologous bone transplantation. Unfortunately, supraphysiological dosage, short half-life time, and extremely high cost significantly limit the applicability of these protein therapies.

Adiponectin, a protein hormone produced primarily in adipose tissue, is secreted into the bloodstream and is very abundant in plasma (5–10 mg/L), accounting for approximately 0.01% of all plasma proteins, compared with many other hormones (Nien et al., 2007). In the last 20 years, the physiological functions of adiponectin in whole-body energy homeostasis have been well documented, particularly the connections with obesity, diabetes mellitus, and atherosclerosis (Li et al., 2009; Yamauchi and Kadowaki, 2013). An interesting finding was the inverse relationship between adiponectin levels and fat mass, which distinguished adiponectin from other adipokines, such as leptin (Yadav et al., 2013). Fat and bone tissues can crosstalk with each other through hormones and cytokines to maintain their balance. In 2004, adiponectin and its receptors, AdipoR1 and AdipoR2, were reported to be present in human osteoblasts (Berner et al., 2004). In addition, the supplementation of culture medium with adiponectin enhanced cell proliferation of mice (Kanazawa et al., 2007). Thereafter, more attention has been garnered on the activity of adiponectin in bone. Numerous studies *in vitro*, *in vivo*, and in clinical settings were carried out to clarify the role of adiponectin in bone physiology (Williams et al., 2009). Adiponectin enhances osteoblast proliferation and differentiation concurrently with the inhibition of osteoclastogenesis *in vitro* (Yang et al., 2019); however, an inverse relationship between serum adiponectin concentrations and BMD was dominantly demonstrated in clinical studies (Richards et al., 2007). The most inconsistent results reported in the literature were obtained from different animal models. The variations are most likely due to the different forms of intercellular signaling, including paracrine effects of adiponectin produced in bone marrow adipocytes, endocrine effects of adiponectin released from white adipose tissue into the bloodstream, and second-order effects from the balance of whole-body energy (Naot et al., 2017).

We particularly focused on the relationship between adiponectin and bone fracture repair in this study, to extend our understanding on the paracrine effects of ADPN through medulla injection. First of all, a facile and efficient approach was designed to obtain purified recombinant human adiponectin. The endogenous glutamine synthetase gene in CHO-K1 cells was selected to be knocked out with the CRISPR technique to speed up the screening process. High purity protein (20 μg/ml) was yielded with our more optimal preparation method. The lyophilized protein powder in glass vials was easy for storage and further usage. The pharmacokinetic behaviors of the derived adiponectin were further evaluated. The C_*max*_ reached a maximum after 8 h *via* medulla injection of ADPN. Longer retention in the bone marrow of ADPN was achieved with intrabone marrow injection, which could provide extended bioavailability. Local administration of ADPN remarkably promoting the growth of callus and bone healing was confirmed in the rat tibia fracture model. With longer duration of local action, ADPN induced hyperplasia, which extended to the non-fractured fibula, indicating, to some extent, the osteogenic ability of local ADPN. This hyperplasia may result from the high dosage of local ADPN and the leakage of the local injection of ADPN to the fibula. This phenomenon warns that the proper dosage and duration of locally administrated ADPN are crucial factors for bone fracture treatment in practice. Biosafety and non-toxicity are of importance for every therapy. Hematology and serum biochemistry tests confirmed the biocompatibility of ADPN for medulla injection.

Local administration of ADPN promoting bone formation in our study mainly results from enhancing osteogenic differentiation. In BMSCs, the ADPN promoted osteogenic differentiation through its receptor AdipoR1 to increase the expression of osteogenic markers ALP, BMP-2, and OCN. In addition, the ADPN also presented the ability of enhancing proliferation up to 30 μg/ml *in vitro*. The addition of ADPN can increase the expression of AdipoR1 in BMSCs considerably, while knocking down of AdipoR1 with ADPN loses their osteogenic ability. Our observations coincide with the results reported in the literature that adiponectin could decrease the number of osteoclasts and improve bone healing *via* the OPG/RANKL pathway (Luo et al., 2006). A potential mechanism for bone regeneration promoted by recombinant human adiponectin was preliminary investigated; however, systemic signal transduction process should be thoroughly examined to unravel the underlying mechanism for the bioactivity of adiponectin in bone regeneration. Heterotopic ossification is the process by which bone tissue forms outside of the skeleton. Heterotopic hyperplasia was observed in the higher-dosage ADPN group in our study, which could have a certain extent of influence on the μCT data, however, it did not alter the conclusion of the ability to enhance osteogenesis and bone regeneration of ADPN. In our future experiment, this phenomenon of hyperplasia is planned to be further investigated.

## Conclusion

In conclusion, we provided a facile method to obtain high-purity and high-yield ADPN for bone fracture treatment, which presented great biocompatibility as well as efficacy for improved bone healing *via* medulla injection. ADPN plays a potential significant role in stimulating the expression of ALP, BMP-2, and OCN at the fracture site through increasing the expression of its own receptor AdipoRl. A dosage of 1 mg/kg of ADPN was optimal to accelerate bone fracture healing in our experimental setting. Our findings demonstrated that local fracture treatment with ADPN could be a useful therapeutic option to shorten healing time and potentially be rapidly translated into the clinics.

## Data Availability Statement

The data that support the findings of this study are available from the corresponding author upon reasonable request.

## Ethics Statement

The animal study was reviewed and approved by the Ethics Committee of the Chinese People’s Liberation Army General Hospital, Beijing, China.

## Author Contributions

YG and Z-KC conceived the ideas for the experimental designs, analyzed the data, and wrote the manuscript. YG, YW, YQZ, LCW, LJW, and YAZ conducted all the experiments and helped with manuscript preparation. CL provided suggestions and revised the manuscript. All authors contributed to the article and approved the submitted version.

## Conflict of Interest

The authors declare that the research was conducted in the absence of any commercial or financial relationships that could be construed as a potential conflict of interest.

## Publisher’s Note

All claims expressed in this article are solely those of the authors and do not necessarily represent those of their affiliated organizations, or those of the publisher, the editors and the reviewers. Any product that may be evaluated in this article, or claim that may be made by its manufacturer, is not guaranteed or endorsed by the publisher.
