# Supplementary material for: Paracrine Effects of Recombinant Human Adiponectin Promote Bone Regeneration
Source: Front Cell Dev Biol. 2021 Nov 1;9:762335. doi: 10.3389/fcell.2021.762335 (PMC8591230; doi:10.3389/fcell.2021.762335)
Supplement: Supplementary file 1 [file Data_Sheet_1.docx]

**Paracrine Effects of Recombinant Human Adiponectin Promote Bone Regeneration**

Yanping Gong^1^,Yang Wang^1^, Yiqing Zhang^2,3^, Liangchen Wang^1^, Lijuan Wan^1^, Yuan Zu^1^, Chunlin Li^1^, Xin Wang^4^, Zhong-Kai Cui^2,3,5,*^

^1^Department of Endocrinology, the Second Medical Center, the People’s Liberation Army General Hospital, National Clinical Research Center for Geriatric Disease, Beijing, 100853, China

^2^Guangdong Provincial Key Laboratory of Bone and Joint Degeneration Diseases, The Third Affiliated Hospital, Southern Medical University, Guangzhou, 510515, China

^3^Department of Cell Biology, School of Basic Medical Sciences, Southern Medical University, Guangzhou, 510515, China

^4^Institute of Orthopedics, the First Medical Center, the People’s Liberation Army General Hospital, Beijing, 100853, China

^5^Bioland Laboratory, Guangzhou, 510005, China

*Correspondingauthor,Zhong-Kai Cui ([zhongkaicui@smu.edu.cn](mailto:zhongkaicui@smu.edu.cn)), Guangdong Provincial Key Laboratory of Bone and Joint Degeneration Diseases, The Third Affiliated Hospital, Southern Medical University, Guangzhou, 510515, China

**Methods**

**Construction of human adiponectin plasmid**

Human adiponectin cDNA was reconstituted according to the specific codon usage based on the modified amino acid sequence of natural human adiponectin (Figure S1). Human IgG-Fc fragment was attached to the Cterminal, and this modified amino acid sequence of natural human adiponectin was listed in Figure S1. cDNA was PCR-amplified, and the band of 748 bp was collected for restriction enzyme digestion. Human adiponectin-Fc-GS plasmid was constructed as follows. The Fc-GS plasmid (Stainwei Biotech lnc, Suzhou, China) was treated with the restriction enzymes, Bgl II and BamH1 (1021A and 1010S, Ensure Biologicals, Shanghai, China), eluted and ligated with the adiponectin fragment to produce the recombinant plasmids, which were then introduced into *E*. coli (GMS12224.1, TOP 10, GENMED, USA) for transformation with the calcium chloride method.The targeting plasmid was collected with the extraction kit (EK007, Ensure Biologicals, Shanghai, China). The recombinant plasmid was treated with two restriction enzymes, Hind III and Xho I (A001303 and B600236,SangonBiotech, Shanghai, China). The derived fragment of ~1434 bp was examined by sequencing.

**Electroporation with the recombinant plasmids and generation of transgenic CHO-K1 cells**

Human adiponectin-Fc-GSrecombinant plasmid was electroporated into CHO-K1 cells, GS gene of which was knocked out. Electroporation procedure was performed as follows. CHO-K1 cells were cultured in DMEM supplemented with 10% fetal bovine serum and 1% penicillin/streptomycin at 37 ^o^C.1×10^7^cells were mixed with 20 μg of adiponectin-Fc-GS plasmids for electroporation procedure. Transfection was carried out in the electronic cup (165-2081, Bio-rad, 0.4 mL, 0.4 cm)under 280 Vfor 20 ms. Cell culture was continued with growth medium after electroporation for 24 h. Screening culture medium containing 50 μM MSX was applied for 14 days. Adiponectin expressing cells were selected and cultured in the CD011 medium supplemented with 1 mg/L insulin, in an orbital incubator for 10 days. Supernatant was collected by centrifugation (10 000 rpm, TG16-WS table model high speed centrifuge, Cence, China). Protein A chromatography column (11-0034-94, GE Healthcare, 10×50 mm) was used to purify the secreted adiponectin-Fc (ADPN). The purity and quantity were confirmed with the western blot method and the ELISA kit (m1061301-3, Mlbio, Shanghai, China), following the manufacturer’s protocol.

**Isolation and characterization of rat bone marrow mesenchymal stem cells**

1. Primary cell extraction: 3-day-old SD rats were euthanized by cervical vertebra division, then immersed into 75% alcohol for 10 min. The bone marrow cavity was repeatedly flushed, and the cell suspension obtained from bone marrow flushing was collected, and the liquid was completely changed after 24 h, then changed every three days.
2. Flow cytometry identification: the 3^rd^ generation BMSCs were identified with marker’s expression, including CD90+, CD34-, CD45- and CD29+ (AF2405, BD, New Jersey, USA). Cell was adjusted to 1x10^6^/mL for detection by flow cytometry (SH800, SONY Corporation, Japan).
3. Differentiation was induced according to instructions (CHEM200013,Saiye Corporation, USA).
4. Cell proliferation test: BMSCs (P3) were counted and inoculated into 96-well plates (5000 cells/well). Different concentrations of ADPN (1μg/ml, 5μg/ml, 10 μg/ml, 30 μg/ml) and the blank control group without drugs were added accordingly. OD values were detected at 12h, 24h, 48h and 72h. (EPOCH Microplate, BioTek, USA) (CCK-8 Cell Proliferation/Toxicity Assay Kit, Tohito, Japan)


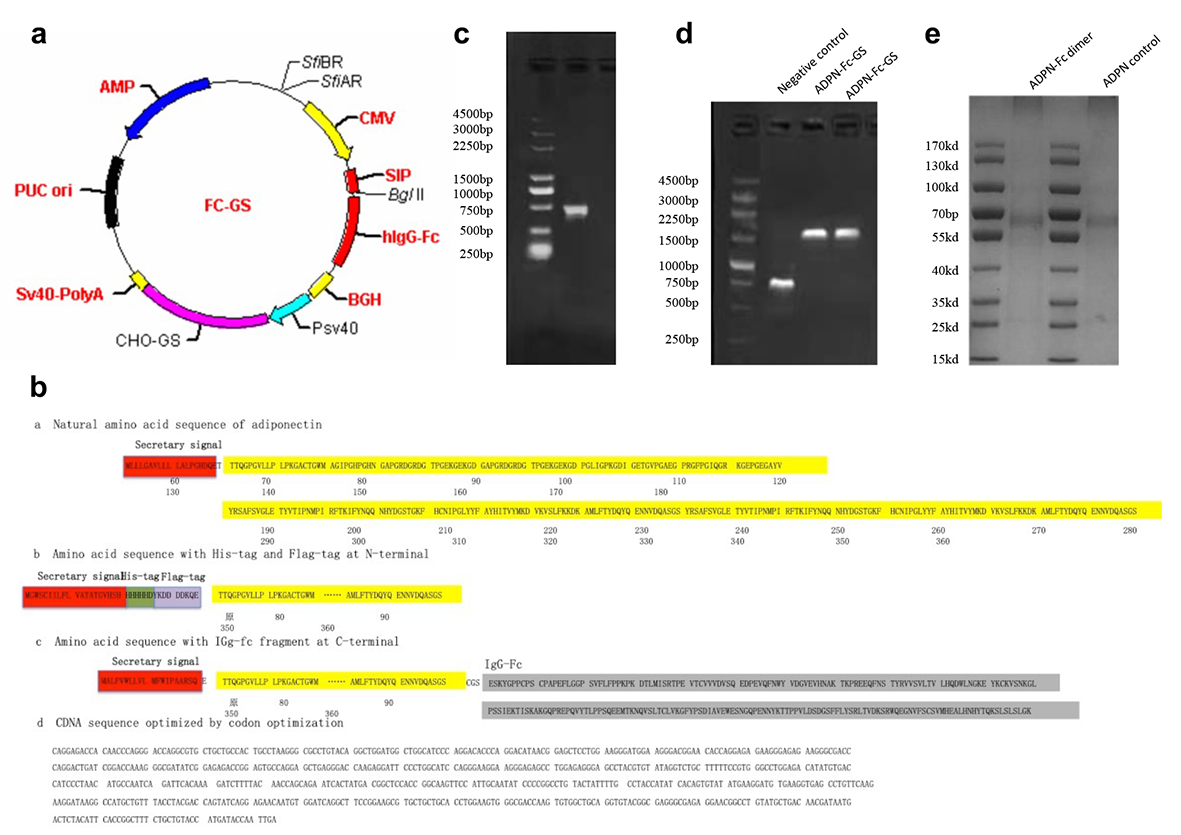


Figure S1. The amplification of ADPNfragment and ADPN-Fc vector construction.

(a) Fc-GS plasmid; (b) Optimized cDNA sequences; (c) ADPN fragmentverified with PCR; (d) ADPN-Fcfragmentverified with PCR; (e) synthesized ADPN proteinverified withSDS-PAGE electrophoresis.


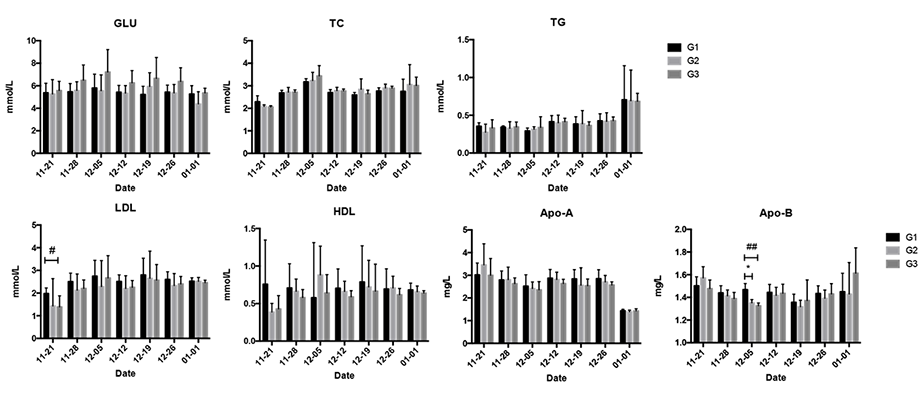


Figure S2. The effects of ADPN on biochemical indices

The blood glucose and blood lipid levels among G1, G2 and G3 groups. **P*<0.05, ***P*<0.01 represented the significant difference between G1 and G2 groups; ^#^*P*<0.05, ^##^*P*<0.01 represented the significant difference between G1 and G3 groups.


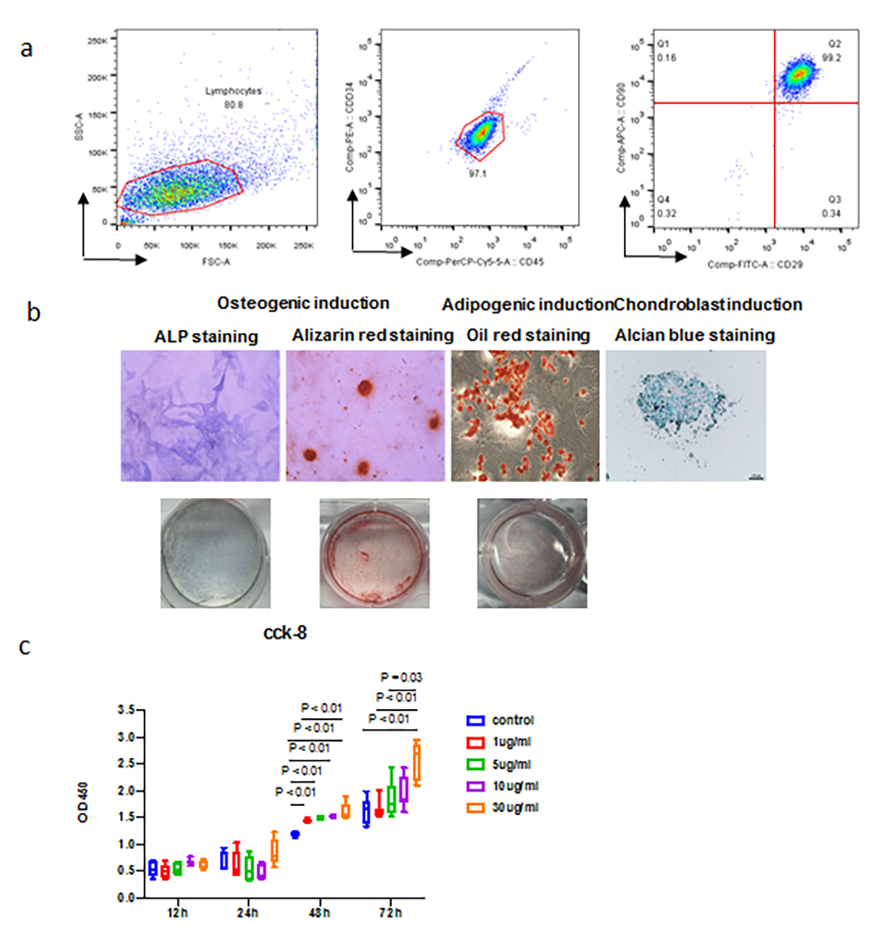


Figure S3. The effects of ADPN on cell proliferation.

(a) Characterization of isolated bone marrow mesenchymal stem cells (BMSCs) with flow cytometry. (b) Differentiation lineage of isolated BMSCs with ALP, Alizarin red S,Oil red and Alcian blue staining to osteogenic differentiation, adipogenic differentiation and chondrogenic differentiation. (c) CCK-8 assay to evaluate the proliferation of BMSCs treated with different concentrations of ADPN (n = 5).
